# Fear, Angst, and the “Startling Unexpected”. Three Figures of Teaching During the COVID-19 Pandemic

**DOI:** 10.1007/s11217-023-09873-9

**Published:** 2023-03-18

**Authors:** Vasco d’Agnese

**Affiliations:** Department of Psychology, University of Campania Luigi Vanvitelli, Viale Ellittico 31, Caserta, 81100 Italy

**Keywords:** Teaching, Covid-19 pandemic, Arendt, Heidegger, Educational community

## Abstract

In this paper, I focus on teachers’ lived experiences during the COVID-19 outbreak. Specifically, I explore the emotional impact the abrupt shift to online teaching had on teachers’ work and life throughout the various phases of the lockdown. I develop my argument by analyzing teachers’ everyday work, using a qualitative approach, and constructing a small-scale empirical study. Philosophically, my attempt is phenomenologically developed and is framed by Heidegger’s and Arendt’s thoughts. Methodologically, my attempt falls within an emerging research horizon that combines educational philosophy with empirical research. Drawing from research data, I argue that teachers’ experience bears witness to a deep modification of the schooling space-time, namely, the horizon in which the very perception of students, themselves, and teaching arise. In the case of teachers experiencing discomfort, fear, and even angst, the totality of their gestures and emotions was, as it were, swallowed by this unknown and disquieting horizon; teachers in distress were experiencing the collapse of any projecting without being freed by projecting itself. In the case of teachers experiencing feelings of fulfillment and even joy, a different horizon was allowed to emerge. Such a different space-time, rather than producing a sense of distance and alienation, created a new intimacy and nearness, which allows for a different attitude toward the topics and the educational community. Students became more sensitive, susceptible to transformation, and open to what was being said. At the same time, relationships were caught in a new web of meanings that transcended them, thus giving birth to an educational experience in its own right. I conclude with some remarks about the building of the educational community.

## Introduction

This study focuses on teachers’ experiences during the coronavirus disease (COVID-19) pandemic. Specifically, I explore the emotional impact the abrupt shift to online teaching had on teachers’ work and life throughout the various phases of the lockdown. I develop my argument by analyzing teachers’ everyday work, using a qualitative approach, and constructing a small-scale empirical study. This research stems from an in-service course I conducted and involves 13 secondary-school teachers (with students aged from 13 to 18) from different disciplines. The study is based on a combination of in-depth interviews, dialogues, and email conversations. Specifically, I collected teachers’ observations and insights when they originated—that is, during the course. Thereby, I reserved myself the opportunity to deepen the most interesting aspects in a manner and at a time that suited the teachers: via email, interviews via Google meet, WhatsApp messages and conversations. Methodologically, my endeavor falls within an emerging research horizon combining educational philosophy with empirical research (d’Agnese 2016; Feinberg 2006; Golding 2015; Hansen 2017; Mejia 2008; Newman and Glass 2015; Santoro 2015; Shuffelton 2015).

Philosophically, my attempt is phenomenologically developed, and is framed by Arendt’s and Heidegger’s thought. Specifically, I will analyze the teachers’ cases drawing from three questions: (a) Heideggerian understanding of “*praesens*,” as the condition upon which the continuity between past, present, and future arises (Heidegger 1982/1927, pp. 306–312); (b) the variations of fear Heidegger points out in *Being and Time*, and his analysis of angst (Heidegger 1996/1927, pp. 131–136; pp. 173–178); (c) Arendt’s thematization of action, beginning, and “startling unexpectedness” of living (Arendt 1998/1958; 1977/1961).

The paper is organized in three parts and a conclusion. First, I expose the research’s context and methods grounding my study. Second, I aim to decipher teachers’ sensations of discomfort and anguish through Heidegger’s thought. Third, I analyze feelings of joy and fulfillment via the work of Arendt. In the conclusions, I summarize my attempt and draw further implications for teaching and education.

## Context and Methods

In December 2019, Wuhan Municipal Health Commission in Wuhan City, Hubei province, China, reported “a cluster of pneumonia cases of unknown etiology, with a commonly reported link to Wuhan’s Huanan Seafood Wholesale Market (a wholesale fish and live animal market selling different animal species)” (Wuhan City Health Committee 2020a). However, at that moment, “no clear evidence of human-to-human transmission” was reported, and “no medical staff infections” were signaled by the Commission (Wuhan City Health Committee 2020b). As a precautionary measure, the State and Provincial Health and Health Commission sent “work groups and expert teams to Wuhan to guide the local epidemic response and disposal work” (Lucey 2021). The rest of the world was largely unaware of what was happening in China. In Italy, we were celebrating Christmas.

On the 20th of January 2020, in a historic press conference, China’s National Health Commission confirmed what had been suspected: the virus spreads from person to person. The new disease was named “Severe Acute Respiratory Syndrome Coronavirus 2 (SARS-CoV-2),” better known as “Coronavirus disease” or “Covid-19” (WHO 2020). After three days, the city of Wuhan started its lockdown. From that moment, news about the virus started circulating. Images of women and men in white suits engaged in sanitizing hospitals, schools, and streets got viral. However, as the virus spread only in China, the common thinking, at least from an European perspective, was that it was limited and precautionary measures were judged unrealistic and exaggerated. On February 3, a group of Chinese researchers published an article on *Nature* titled “A pneumonia outbreak associated with a new coronavirus of probable bat origin” (Zhou et al. 2020).

In February 2020, in the North of Italy, precisely in Cologno, Lombardy, was identified the erroneously labeled “patient zero.” It was just the beginning of the first devastating wave of the virus in South Europe. Starting Sunday, March 8, 2020, the Italian government announced the first nationwide curfew. However, the news were conflicting and feelings about the situation were mixed. Adults were torn between fear of an unknown threat and hope that the problem would be solved soon. “It’s just another flu epidemic” —this is the sentence someone repeats as a prayer.

The TV announced school closing for two weeks. Children–as much as strange and tragic it may seem *now*–celebrated. However, two weeks were not enough. Very soon the situation worsened. The lockdown, or, as the authorities called it, “social distancing,” remained our best defense against the virus. Girls, boys, and children were deprived of school, sports, and any other possibility of contact–apart from online interaction. Doctors and nurses were helpless, and every day the report of death count increased. Economy collapsed. No one was safe; no one knew how long the nightmare would last. However, we also witnessed a manifestation of resilience. Banners were displayed on balconies: “We’ll be fine.” That was the mantra repeated over and over again in those days.

School lessons came online in mid-April. As parents of two sons, my wife and I received an email informing us about the Google platform and the new school organization. Thus, thanks to the effort of teachers, principals, and families, girls, boys, and children began, although just online, to interact again. It was a breath of air. However, it put a burden on teachers: they had to learn quickly how to conduct online classes, take care of students in this situation, organize homework, assessments, and all the other tasks associated with teaching. In my personal experience (but, I believe, it was very common), everything had to be done while children were struggling with online teaching and relatives were sick, without having the opportunity to see or touch them.

In this context, a principal I knew, called me to a training session for teachers. Knowing the school and the dedicated teachers that work there, I accepted their invitation. Working with teachers is one of my favorite things and, at that moment, it seemed like a way to help.

The subject of the course was really broad, namely, “Democracy, Technology, and Inclusion.” The group consisted of 30 secondary school teachers (with students aged 13 to 18) from different disciplines, and the course was based on voluntary participation.

I began my course—one three-hour session per week for two months—and, as I typically do, I gave teachers time and space during the online class to share their questions, perspectives, experiences, and counterarguments. Right from the start, I could perceive a desire, even an urgency, to speak up and share doubts, observations, and experiences. Very soon, in the space devoted to discussion, such observations and experiences began to revolve around the situation they were facing: being online before their students with no training, while the world around us seemed to collapse. In the beginning, while acknowledging the urgency they felt—more or less, I was facing the same situation—I attempted to bring the discussion back to the subject of the course. Sometimes teachers, when discussing an issue, slowly slide onto other topics. Therefore, I am prepared to bring the discussion back to the core arguments, avoiding that the course may turn into a support group–as valuable as support groups can be. However, the more I tried to discuss the course topic, the more I scrolled through the slides, and the more I felt how futile and even phony my effort was. The teachers’ minds and hearts were elsewhere, and rightly so. My task at that moment was not so much to present Dewey’s view of democracy in terms of instruction and technology, but rather to give voice and legitimacy to their feelings and experiences, creating a space for the restoration of unity that many of them were demanding. This is what I tried to do.

I abandoned my program and centered my course on “Engaging with the unexpected.” Meaningful experiences emerged, and the sharing of perspectives created a space for teachers to deal with the situation they were facing. Of course, I do not want to overstate my attempt, either in terms of helping teachers or in terms of revealing my research. In other words, the chaos, discomfort, powerlessness, and anxiety were not resolved. We merely created a space where feelings and fears were translated into language. As for my study, as Laura Duhan-Kaplan (1998) has adequately pointed out, when exploring personal experiences (one’s own or others’), we should not attempt to generalize these experiences, nor should we overstate them, as if feelings of anxiety or joy, such as those experienced by the teachers I met, were more valuable or important than other experiences. Therefore, I just hope that by sharing my experiences I can shed some light on the tearing and even limit situation that teaching can be.

Before addressing methodological questions, a clarification about the research background is needed. Throughout my years of experience as a teachers’ educator, I have often encountered groups of teachers who are passionate about their profession. Often, these teachers—given their deep commitment—were radically exposed to difficulties, frustrations, and failures; however, they could also feel deeply the joy, passion, and possibilities stemming from working in the education field, even more than others. During the discussions presented during the courses I conducted, these teachers would often share insightful considerations and reveal personal feelings related to different aspects of teaching; these included teacher–student relationships, curriculum, professional judgment, and the consequences of their passion and commitment. I would always take note of their interventions, and sometimes, at the end of the course, given the quality of interactions, I would stage research settings to gather such insights systematically. However, at times, when comparing gatherings from experimental settings with the rough material that emerged during the courses, I felt that something was lost. On those occasions, I was left with a subtle sadness and desire to recover the lost insights.

I cannot, of course, ignore the possibility that such a difference was due to a failure on my part to create an adequate experimental setting. However, I do believe that something more was at stake. Simply put, the matter teachers shared during the open discussions during the course was something too elusive and difficult to handle to reproduce in a dry experimental setting; the urgency they felt to communicate their deep feelings and insights as part of the matter they were sharing, something that is hardly replicable on demand. Indeed, deep insights and emerging feelings are not at our disposal. Therefore, if we wish to shed light on the actual lived educational experiences, we must carefully listen to when and where such experience arises.

Considering the above, for this study, I decided to proceed in a different manner: I would collect teachers’ observations and insights the moment they originated, reserving myself the opportunity to deepen my investigation of the most interesting aspects of their statement in a manner and at a time teachers preferred, via email, interviews via Google meet WhatsApp messages, and in-person conversations. I, of course, informed the teachers of my research purposes and experimental setting from the very beginning.

Therefore, when something revealing came to light, I invited the teacher involved to deepen their experience, focusing on their impressions and feelings while exploring the possibility of further expanding their insights. In some cases, I also asked the teachers about an image or metaphor that could better convey the sense of the experience being shared.

From the group of 30 teachers involved in the course, I first chose 13 teachers for my research purposes. Such a selection was based on the meaningfulness of their insights related to my study and their willingness to share and inquire about their experiences further. A second selection was made in the reports of their findings; here, I selected reports from 5 teachers: Silvia, Luigi, Gennaro, Davide, and Giovanna. By prior agreement, the interviews were anonymized, and teachers’ names are pseudonyms.

From the outset, it was clear that difficulties, discomfort, and even suffering were important aspects of teachers’ emotional state. “Helplessness,” a “permanent sense of warning,” “loss of oneself,” and even “anguish”—as two teachers described their emotions—were the prevalent mood related to the online teaching experience. However, these were not the only feelings experienced by the teachers I met. Along with these feelings—and to my surprise, admittedly—some teachers also spoke of a “wholeness,” a “fulfillment never experienced,” and even “a deep sense of joy” and an “unknown freedom” in encountering their students online.

How should one make sense of such a diversity of emotions? Of course, one might argue that this question makes little sense, as the diversity of sensations relies on the diversity of individuals and approaches—and, in a basic sense, this is true. One might even argue that teachers who experienced fear and angst when going online could not teach, for teaching also involves facing unexpected situations. However, I contend that much more was happening within teachers’ emotional horizons and lived experiences. When I listened to teachers and attempted to understand and unravel their emotions, I felt a common root: all those diverse and even opposite feelings were connected to a deep ethical engagement with students and the profession and the struggle to face an unknown situation in an unknown manner. Otherwise stated, it was the entanglement of the unknown space in which teachers were thrown with their commitment and responsiveness to students that caused such a deep and diverse bunch of emotions.

Methodologically, given the nature of this work, I can place this study within an emerging research framework combining educational philosophy with empirical research, where empirical should better be understood as “bearing witness” (Hansen 2017). The phrase by David Hansen well conveys a sense of stepping back, waiting, and listening, one “capable of being in uncertainties, mysteries, doubts, without any irritable [impatient] reaching after fact and reason” (Keats quoted in Todd, 2015).

Such a step back means to be faithful to matter, which I would say is both heavy and ephemeral. Thus, “engaging firsthand what is happening in an educational setting [and…] reflecting upon it through a philosophical lens” serves to illuminate “aspects of current policy, practice, and research that might otherwise remain in the shadows” (Hansen 2017, p. 9). Such an engagement also has a side effect: rendering philosophical assumptions and inquiry more concrete (Lewis 2017). No doubt, as human beings, we live with, through, and by our thoughts, and, at least from Dewey onward, we know all too well that to think is to risk and act (Dewey 1910, p. 19). Philosophical inquiry, then, is a living endeavor. However, just for the depth of philosophical thinking we should use it directly, so to speak, for the deepening of concrete experiences in teaching and education. On the other hand, as Feinberg rightly points out, “[e]thnography provides powerful tools with which to understand the problems of people, as experienced in everyday life. Yet relatively few philosophers engage ethnographic research and even fewer incorporate its methods into their own work” (2015, p. 151).

In educational research–or any other research, for that matter–certain aspects may be silenced, and others may not gain “theoretical” or “methodological dignity.” Transparency, warrantability, external validity, and reliability do not always work in a benign way. Of course, this is not a call to obscurity, which would be senseless, if not ridiculous. This is a call to live, educate, and inquire with and by the “mystery, doubt, and half-knowledge” of the world in which human beings live (Dewey 1980/1934, p. 34) for in experience “the distinct and evident are prized… but… the dark and twilight abound.” (Dewey 1929/1925, p. 20) Sometimes we must put a light on what is dark, illuminating it. Sometimes such a light has to be put aside to leave the twilight intact, touching it as little as possible, thus exercising what Hansen defines as “an ever-deepening attentiveness” (2017, p. 10) to the voices of persons engaged in education, to the nuances of such voices. It is a handcrafted, fine-grained work that consists of silence, listening, and waiting. We have to wait to feel and understand the signals and words that appear in context. In a Heideggerian vein, we must leave the words and statements in their being.

Philosophical reflection may thus work as a companion while “qualitative research and philosophy are treated as similar intellectual undertakings and can therefore be thoroughly integrated.” (Shuffelton 2015, p. 138) Philosophizing as a kind of witnessing can thus fulfill the task of deepening concrete experiences and at the same time gaining concreteness from such an experience. Philosophizing may thus offer “new descriptions of phenomena that provide alternative explanations for teachers’ experience” (Santoro 2015, p. 173) enlarging such experiences, giving legitimacy to the fact that teaching is not such a straightforward matter, but something residing in the encounter between teachers’ ethics and reflective engagement and the educational endeavour itself. Beyond and behind what has been framed as the “[s]kill-ful teacher” (Brookfield 2015), and “teaching effectiveness” (Ronfeldt 2015), there is a world of messiness, joy, and struggle that may display its meaning through our hearing. The method that emerges from such scholarship encourages researchers to delve deeply into things, however confusing and confounding they may be.

Perhaps one of the most beautiful expressions of this stance lies in the Heideggerian work, *A Letter to A Young Student* (1950/1971). Heidegger begins the letter with a young student’s question, “Whence does thinking about Being receive (to speak concisely) its directive?” (ibid., p. 181). Heidegger responds as follows:

“[t]o think ‘Being’ means: to respond to the appeal of its presencing… This thinking… is only a possible occasion to follow the path of responding, and indeed to follow it in the complete concentration of care and caution toward Being that language has *already* come to” (ibid., pp. 181–184, emphasis in original).

Responding to “the appeal of… presencing” places human beings into a situation of radical dependency: neither in acting nor in thinking we are ever at our disposal. We must attend to such a matter with “care and caution” (ibid., p. 184), recovering a stance toward the world which allows for new meanings to emerge.

To begin my analysis of teachers’ emotions and experiences, a few words about my choice to read such emotions and experiences through Heidegger and Arendt are necessary. My choice for Heidegger lies in a quality of his thought well-described by Magrini: “Rather than philosophizing, Heidegger is concerned with embracing poetic, ‘meditative’ thought” (2012, p. 502). Such a quality has already been noted by Standish (1997), who also spoke about the humility that can stimulate Heidegger’s thinking. According to Standish, such humility “needs to be linked with a sense of the precariousness of the human situation. Beyond this, humility is reciprocally related to a sense of mystery or wonder” (1992, p. 22). Such a meditation in humility and the sense of mystery and wonder related to such a stance are very much in line with my attempt, just as Heideggerian interpretation of subjectivity based on a concrete sense of living. As Moran noted, Heidegger understood “subjectivity in a way that… conveys its sense of living, temporal historical existence… with all its connotations” (2014, p. 493). Additionally, as far as I can see, no philosopher has gone as deep as Heidegger in analyzing fear and angst, namely, two of the feelings emerging from this study.

What struck me about Arendt is the sheer inversion of the (Western) conception of identity we may find in her work. Rather than relying on thought or some other theoretical virtue, Arendt saw identity as something that arises from one’s actions and speech. For Arendt, public space constitutes the private, or rather there is no private space disjointed by the public (1998/1958)- I shall return to this further. The relational dimension and, thus, the political dimension permeate human existence to the core. Moreover, Arendt’s commitment to uniqueness is something we, as educators, should always keep in mind. As Arendt wrote, “there are no general standards to determine our judgments unfailingly, no general rules under which to subsume the particular cases with any degree of certainty.” (2003/1978, vii) Judgment, and thus research, in Arendt, is always a personal commitment (Todd 2007). Additionally, my choice was also due to Arendt’s devotion to narrative as something that can capture a sense of freedom and life. According to Kristeva, Arendt was:

“A fervent admirer of the ‘narrated life’, of *bios-graphie*…Thus, the possibility of representing birth and death, to conceive of them in time and to explain them to others – that is, the possibility of narrating – grounds human life in what is specific to it, in what is non-animal about it, non-physiological… Arendt rehabilitates the *praxis* of the narrative. Challenging the remoteness of the poetic work, only action as narration, and narration as action, can fulfil life in terms of what is ‘specifically’ human about it. This concept… links the destinies of *life*, *narrative* and *politics*” (2001, p. 6, emphasis in original).

Apart from that, I would highlight how the study of philosophy and philosophizing is always a very personal matter. While Arendt’s insight that our identity is revealed not in our thoughts but in our actions and speech has enlightened me, I cannot help but believe that as researchers we live with what we read, think, and study, and that our experiences frame such a studying and are shaped by it. Setting aside any theoretical correspondence or similarity between the material analyzed and the means of analysis, I fear that my choice is based fundamentally on my engagement with philosophy. In addition, I cannot exclude any other interpretation, and accordingly one could choose any other eminent philosopher to shed light on the words and experiences. Moreover, it is in the nature of the study that the use of other thinkers would have changed the interpretation of the teachers’ experiences and perhaps even the selection of statements, emotions, and experiences worth analyzing. Thus, my hope is just that the comparison with Heidegger and Arendt will be considered useful. In what follows, I will try to convey this choice as plausible. This means, I believe, bearings witness through philosophy.

## *Praesens*, Fear, and Angst

In this section, I aim to decipher teachers’ sensations of discomfort and anguish via Heidegger’s thought. The section is divided into three steps that are committed to (a) reporting certain significant excerpts of teachers’ conversations and interviews; (b) analyzing the question of *praesens*, (Heidegger, 1982/1927, 306–312); and (c) connecting teachers’ observations with Heidegger’s analysis of fear and angst (Heidegger 1996/1927, pp. 131–136; pp. 173–178). I start with the teachers’ interviews.

### Teachers’ Interviews

The material I collected during my research is more extensive than what can be reported. As stated in the *Introduction*, the former group of 30 teachers participating in the course has been restricted to 13 teachers due to both teachers’ willingness and the meaningfulness of their experiences given my aims. The section is divided into three parts. In the first part, I report statements from three teachers regarding anxiety, fear, anguish and a deep sense of helplessness arising from their experiences. In the second part, I analyze Heideggerian understanding of *praesens*, attempting to relate my analysis to teachers’ experiences. In the third part, I focus on fear and angst. I begin with Silvia’s, Luigi’s and Gennaro’s reports.

### Silvia’s Report^1^

“During the lockdown, I woke up with a weight on my chest, and a sense of permanent warning … When my class was about to start, I would reach for my computer, go online, and begin the lecture. Sometimes it was not that bad. I could see their faces and I could imagine what was going on in their minds… But there always was a gap I couldn’t fill. It was something like a permanent lack I was unable to compensate for, and I felt guilty for that, although I knew it was not my fault. This awareness didn’t help me make sense of my work.”

### Luigi’s Report^2^

“The Third B [Luigi’s class, ed.] had always been a hard one. But online, it was even worse. When you see them and can interact face to face, you can engage their attention, present examples, use irony, and reproach effectively. When you are on Meet, none of these things makes any sense. Any reproach becomes comical…Within two weeks, I found myself in a spiral…It was a total nightmare. Sometimes I was so panicked that I had to break up my lesson. I felt like I was going crazy…I had to call my wife just to speak with her and break that sense of anguish.” When asked about an image conveying such a sense of angst, Luigi reported about “an empty room, all dark, with my voice banging around… A total collapse”

### Gennaro’s Report^3^

“When going online, my prevailing feeling was that the class didn’t follow me… I was speaking in front of that screen with no evidence that someone would listen… I feel the right word for my experience is fear… It’s difficult to explain, but I felt as if the whole situation was an actual threat, and I couldn’t do anything to stop it ... At times, I couldn’t think, for if I did, I would have been paralyzed, in panic. I would just go on with my lesson, trying to not think.”

### Praesens

In Chapter One, Part Two of *The Basic Problems of Phenomenology*, when explaining the “temporal interpretation of being as being handy,” Heidegger introduces the question of *praesens* as the “horizonal schema of the ecstasis of enpresenting” (1982/1927, pp. 303).

Heidegger furnishes the first indication of the meaning and function of *praesens* when stating that *praesens* is the condition upon which “presence and absence” present themselves to us (ibid., p. 304). As for other questions–time, for example–Heidegger is concerned with delving into the very fabric of one’s being-in-the world. Thus, how is it that Dasein can perceive something as present or absent? Heidegger makes clear that before perceiving something as present or absent, human beings—Dasein, in his understanding—are entangled in the world in such a way as to be able to deal with a more fundamental structure. This ability is the original structure of *praesens*. Otherwise stated, *praesens* is the very texture upon which the world, others, and things—including emotions—may be encountered, and the condition of the possibility of any understanding.

Importantly, in being the condition upon which we may encounter and perceive what is present and what is not, it also is the condition of possibility of both the “beyond itself” (ibid., p. 304) and the present, the very now. Even enpresenting, in Heidegger’s words, “understands itself as such upon *praesens*.” (ibid., p. 307).

Thus, when analyzing the statements of teachers I met with, we may note that their words bear witness to a deep modification of the texture of their being in the world, namely, the structure of *praesens*. In other words, the horizon in which the perception of the students, themselves, and the teaching takes place changes dramatically. Any word or silence, any gesture or act, stands on the basis of such a modification. Even one’s commitment and projecting come to be felt upon such a different horizon, by means of which “anything like existent commerce with entities handy and extant becomes possible” (ibid., p. 309). This is also due to the relationship between *praesens* and temporality: given that Heidegger states that *praesens* is “already *unveiled* in the self-projection of temporality” (ibid., p. 309, emphasis added), such a deep structure goes hand in hand with temporality itself. The way in which temporality is given to teachers—or, in a Heideggerian vein, how temporality temporalizes itself in teaching—relies on *praesens*, too. The temporality of the teachers, based on the accounts of Silvia, Gennaro and Luigi, becomes unbearably dense, but not in the sense that it is full of events. Quite the opposite: their teaching routine involved the same feeling every time. Remembering Heidegger’s understanding of attunement as “not some being that appears in the soul as an experience, but the way of our being there with one another” (1992/1929–1930, p. 66), Silvia’s, Gennaro’s and Luigi’s teaching were always attuned in the same way, no matter where and when they taught.

Considering this, what happens to *praesens*, to such a fundamental structure when one is “paralyzed, in panic” and cannot think at all? (Gennaro’s report) What if one is “so panicked… to break up” the lesson (Luigi’s report)? What if teachers in distress were to experience, using Luigi’s powerful words, “a total collapse of time?” In such a condition, one cannot think and act—to say nothing of projecting. Relationships are blocked, as they are perceived as threatening. The totality of gestures and emotions teachers feel and enact is under the shadow of this curtain. Hoping, listening, projecting, awaiting, looking at, talking, hanging back, caring—the whole patrimony of comportments teachers enact in daily classroom activity—is, as it were, swallowed by this unknown and disquieting kind of *praesens.* What Heidegger calls the “intentional comportments toward the futural” (1992/1928, p. 204) undergo the sway of this new consistency of time-space, which is, as discussed further, permeated with feelings of alarm, fear, and angst.

### Heideggerian Analysis of Fear and Angst

In this third step, I recall Heidegger’s analysis of fear and angst and relate this analysis to the teachers’ experience. We begin with Heidegger’s phenomenological analysis. I will quote two passages from *Being and Time* that describe fear and angst, respectively, and then provide my comment.

“The phenomenon of fear can be considered in three aspects. We shall analyze what we are afraid of, fearing, and why we are afraid […]. That before which we are afraid, the ‘fearsome,’ is always something encountered within the world, either with the kind of being of something at hand or something objectively present or *Mitda-sein* […]. What is feared has the character of being threatening. Here several points must be considered.

1. What is encountered has the relevant nature of harmfulness. […]
2. [H]armfulness […] comes from a definite region. […]
3. As it approaches, harmfulness radiates and thus has the character of being threatening.” (1996/1927, pp. 133).

Approximately forty pages on, in Chapter VI, Heidegger describes angst as “[t]he fundamental attunement” and the “[e]minent disclosedness of *Da-sein*”:

“That about which one has *Angst* is being-in-the-world as such […]. What *Angst* is about is not an innerworldly being. […] The threat does not have the character of a definite detrimentality, which concerns that is threatened with a definite regard to a particular factical potentiality for being. What *Angst* is about is completely indefinite […]. The totality of relevance discovered within the world of things at hand and objectively present is completely without importance. It collapses. […] The fact that what is threatening is *nowhere* characterizes what *Angst* is about […] But nowhere does not mean nothing […]. It is so near that it is oppressive and stifles one’s breath—and yet it is nowhere […]. In *Angst*, the things at hand in the surrounding world sink away, and so do innerworldly beings in general.” (ibid., p. 176).

When comparing Heidegger’s analysis with the teachers’ experience, we note that the teachers’ words and emotional qualities bear witness to both fear and angst. Gennaro, Silvia, and Luigi all encountered the fearsome within the world in a definite context; moreover, it is also true that a “harmfulness radiates and thus has the characteristic of being threatening.” What is problematic in defining their experience as just one of fear is that harmfulness—rather than aiming “at a definite range of what can be affected by it”—extends to the whole professional and even personal experience. Even Gennaro’s and Silvia’s sense of self when being in education was jeopardized by the profound change. Furthermore, while Gennaro, Silvia, and Luigi encountered fear in the realm of “innerworldly beings,” they clearly experienced a sinking away of the world: Gennaro’s inability to think, his paralysis and panic, bear witness to the fact that being in teaching, as such, was the source of his angst. What Gennaro experienced while being online was a total breakdown of intelligibility; in his experience, the ecstatic projection of *Da-sein* felt down. Otherwise stated, both acting and thinking collapsed, and the world itself pulled back. Yet, much like fear, such angst comes from a well-defined region: being online with his students.

Similarly, Silvia speaks of “a sense of permanent warning.” As I understand it, the sensation is a combination of alarm, as the precursor of fear, and something very similar to the “nowhere” and “nothing” of angst. It is an indefinite yet stinging sense of danger that looms at all times. In this case, too, what Silvia experienced is a collapse of the former *praesens* and the irruption of a new kind of *praesens*; as Heidegger writes, we cannot lose *praesens*, for *praesens* is simply what enables the possibility of experiencing something (1982/1927, p. 310). When teaching online, Silvia felt severed from both her students and her profession. Even the bodily sensations Silvia experienced were akin to those provoked by angst: the “weight on the chest” she felt when getting up is akin to the feeling described by Heidegger, one which “stifles one’s breath.” Yet, in this case, too, the threatening feeling comes from a well-defined region and activity.

However, among the experiences reported, the one which best displays the features of angst was perhaps that of Luigi. He spoke about a “total collapse,” a sense of panic that forced him to break off the lesson and call his wife. Even the image he found “an empty room, all dark, with [his] voice banging around” is a powerful representation of a pervasive—yet unfathomable—feeling. Yet, in this case too, Heidegger’s description is simultaneously apt and limited. When speaking of angst, Heidegger states that “[w]hat *Angst* is about is completely indefinite” and “[a]ngst does not know what it is about which it is anxious.” (1996/1927, p. 176) However, this was not the totality of Luigi’s experience. Luigi was aware of where angst came from, and could exactly locate its source; and yet such a root—being online with students—provoked a sinking away of the world or, in Heidegger’s terms, of the “innerworldly beings in general” (ibid., p. 176). Luigi was not oppressed by this or that problem of teaching; rather, Luigi was oppressed by the possibility of teaching as such. To be more precise, he realized the impossibility of his being a teacher with teaching being understood as not a profession, but a way of being. That which was threatening to Luigi approached from a well-defined region—and yet, as Heidegger states, it also was “nowhere.”

It is important to note that Luigi was forced to move on with a lecture that did not make any sense: he was forced to move on with a lecture that projected a loss of meaning into his body and pushed him to a limit-possibility—the death of his project. Drawing on Heidegger’s analysis of death, we could suggest that Luigi was experiencing “the possibility of an impossibility” (ibid., p. 330). His project, namely teaching, was impossible to pursue in the situation he found himself in; yet, the project was there, hideously disfigured and causing him to sink in angst. By drawing from Thomson’s interpretation of Heidegger, Luigi was experiencing “a global collapse of […his] identity-defining life project.” (ibid., p. 331) However, what rendered Luigi’s experience so peculiar was that Luigi was experiencing the collapse of any projecting, without being freed by projecting itself. At this point, the analysis of the question of projecting may be helpful.

In *Being and Time*, reminiscent of Kierkegaard, Heidegger speaks of Dasein regarding projects and possibilities:

“Project is the existential constitution of being in the realm of factical potentiality of being. And, as thrown, Dasein is thrown into the mode of being of projecting. Projecting has nothing to do with being related to a plan thought out, according to which Dasein arranges its being, but, as Dasein, it has always already projected itself and is, as long as it is, projecting… Furthermore, the project character of understanding means that understanding does not thematically grasp that upon which it projects, the possibilities themselves. Such a grasp precisely takes its character of possibility away from what is projected, it degrades it to the level of a given, intended content, whereas in projecting project throws possibility before itself as possibility, and as such lets it *be*. As projecting, understanding is the mode of being of Dasein in which it *is* its possibilities as possibilities.” (Heidegger 1996/1927, p. 136, emphasis in the original).

This is a fundamental passage for understanding Heidegger’s thought. Here, Heidegger emphasises how Dasein must always be understood as already projecting: “Dasein is thrown into the mode of being of projecting”; that is, the only “structure” from which Dasein cannot escape is the projection. Such projecting, as the fundamental feature of Dasein, is not a choice: we are not free from projecting ourselves. Thus, Dasein must always bear the responsibility of being thrown “into the mode of being of projecting”; the condition of projecting always dominates Dasein, whatever Dasein thinks or makes. For this reason, Luigi could not be exempted from projecting. He had to endure that a project collapsed while he was standing in front of his students. In this sense, he was experiencing the collapse of *praesens*, of the connection with temporality and worldly horizons, without the possibility of being released by the *praesens* of teaching. Otherwise stated, Luigi, as a committed teacher, passionately chose the *praesens* and projecting he had to endure. While human beings are thrown into “the possibility of an impossibility” which is death, Luigi—committed to his students—was the author of his own “possibility of impossibility.”

## Freedom, Joy, and Fulfillment

Thus far, I have aimed to decipher teachers’ experiences of fear and angst through Heidegger’s thought. In this section, I analyze a different range of emotions emerging from online teaching—feelings of “wholeness,” “fulfillment,” and “joy.” I discuss this point by drawing from Arendt’s questions of action, beginning, and “startling unexpectedness.” Like the previous section, this section comprises three steps, committed to (a) reporting significant excerpts of teachers’ interviews; (b) analyzing Arendt’s questions of action, beginning, and “startling unexpectedness”; and (c) analyzing teachers’ emotional experience while elucidating the building of the educational community. I begin with teachers’ experiences.

### Teachers’ Interviews

#### Davide’s Report^4^

“During the lockdown, I had to leave my study to make room for my son; thus, I worked in my bedroom. In the beginning it was kind of awkward: in the background you could see the bed and clothes lying all over the room… I managed to put up a background from Google tools, but for some reason it didn’t always work. After a while, I resolved to accept the situation, and began to joke about my mess… I began to feel a kind of coziness in that situation. I mean, not just my own coziness in being in my bedroom with a cup of coffee… I felt a kind of shared, common coziness… everyone in their bedroom or living room, with their cup of coffee, chocolate, juice or whatever… So, we decided to have coffee-break together. Some students would prepare toast, others would even cook… It was strange, for we were separated, far from each other, and yet we were closer than ever. At some point, I thought it would have been nice playing some music in the background when teaching. Classical music, rock, pop… we all felt a kind of togetherness in that strange situation… New connections began to emerge… I mean, connections among topics, among students, between me and them, between them and such new topics… At last, I felt students speaking with their voice. I mean, they began reading poetry, making personal comments on authors they studied… Everything was new in those months.”

#### Giovanna’s Report^5^

“At the beginning it was bewildering… before the screen, speaking to everyone as always, but in a very different way… You couldn’t know whether your students understood the topics… it was even difficult to understand whether they were there to listen. In some way, it was embarrassing. So, as a way out, I began to concentrate on the topic being explained.” When asked about her focus, Giovanna said: “I intentionally decided to exclude any thought about whether students were there and what they understood about the lesson. I began to delve into my topic. Should I find an image, I felt as a researcher at a microscope...” At this point I asked if one could label her lesson as a kind of study. Giovanna told, “yes, I was studying, but I was studying in front of my class!” When asked about the outcomes of her gesture, Giovanna said: “Unexpectedly, students were interested in such a strange lecture… They were following me… They began to interact and delve into the topics… I discovered a new way to teach… I experienced an unknown freedom by teaching online, from my bedroom.”

### Action and “Startling Unexpectedness”

The questions of action, beginning, and “startling unexpectedness” (Arendt 1998/1958; 1977/1961) are central to understanding Arendtian oeuvre. They are not just extensively addressed by Arendt throughout their career; they also form the core from which many significant questions unfold. Through them, questions on humanity, natality, and existence connect with and illuminate one another in a web of implications that enriches the meaning of these themes, thus presenting an open and powerful conception of life. However, with my end in view, far from presenting a fully-developed account of these themes, in this step, I will present some features which may help shed light on the excerpts I selected.

In *The Human Condition*, when discussing the common ground upon which action and speech should be understood, Arendt states that through action, human beings

“…distinguish themselves instead of being merely distinct.” Action is the way “in which human beings appear to each other, not indeed as physical objects, but *qua* men. This appearance… rests on initiative, but it is an initiative from which no human being can refrain and still be human.” (ibid., pp. 176–177).

Through this statement, Arendt overturns the meaning of action, with far-reaching educational consequences. For Arendt, newness *and* human beings come into the world by means of action. From an Arendtian perspective, in fact, action takes place “between men,” and only through action may human beings’ “specific, objective, worldly interests” arise. These interests, in Arendt’s words, “constitute, in the word’s most literal significance, something which *inter-est*, which lies between people and therefore can relate and bind them together.” (ibid., p. 182) For Arendt, action is strictly related to the ability human beings have to take upon themselves the responsibility, burden, and bearings of their distinctness. Without denying the well-known Arendtian concern about any attempt to find the essence “of human existence,” (ibid., p. 9), we may infer that, in Arendtian terms, action is essential to being human, and that taking “initiative” is, for Arendt, something that characterizes human beings. By means of action, human beings come into the world and accomplish the newness to which they are destined. How and when such an initiative should be taken and enacted is left open and undetermined by Arendt. However, enacting our own initiative is what makes us human. As Birmingham aptly noted, according to Arendt, action is grounded in the “archaic and unpredictable event of natality.” (2006, p. 3).

A first pedagogical result of such an understanding is that educational spaces are conceived as spaces in which human life takes shape, and not only as places where one acquires knowledge and competencies to face and cope with the world. For human beings, living is established moment by moment *while* being together; living and meaningfulness come into place only in the dimension of togetherness. According to Arendt, “Men in the plural, that is, men in so far as they live and move and act in this world, can experience meaningfulness only because they can talk with and make sense to each other and to themselves” (Arendt 1998/1958, p. 4). The failure to recognize such a public dimension of meaningfulness results in “modern world alienation,” that is, the “flight from… the world into the self” (ibid., p. 4). Thus, both the very reality of the world and things and the reality of ourselves spring from the presence of others: “The presence of others who see and hear what we see and hear assures us of the reality of the world and ourselves” (ibid., p. 50). To clarify, human beings’ uniqueness comes from the fact that one reveals who she is to others through her actions and speech, while at the same time revealing who she is to herself. Therefore, we cannot know who we are until such a revelation takes place.

We should also note that, for Arendt, being human and being a beginner performing their own beginning through action in a public space are one and the same thing. This is because the significance of action is twofold. First, action is central to understanding and acting in freedom. As Arendt states, human beings “are free… as long as they act, neither before nor after; for to be free and to act are the same” (Arendt 1998/1958, p. 153); second, action is essential to addressing and producing the new, and education reflects exactly such a broad and persistent commitment to newness. Through their capacity to act, human beings fulfill the promise contained in their birth: “The new beginning inherent to birth can make itself felt in the world only because the newcomer possesses the capacity of beginning something anew, that is, of acting.” (ibid., p. 9) Women and men are free because they are “a beginning.” (ibid., p. 167).

Here, I focus on the relationship between action, and uniqueness and plurality:

Action… corresponds to the human condition of plurality, to the fact that men, not Man, live on the earth and inhabit the world… Action would be an unnecessary luxury, a capricious interference with general laws of behavior, if men were endlessly reproducible repetitions of the same model, whose nature or essence was the same for all and as predictable as the nature or essence of any other thing. Plurality is the condition of human action because we are all the same, that is, human, in such a way that nobody is ever the same as anyone else who ever lived, lives, or will live. (Arendt 1998/1958, pp. 7–8)

In this passage, action is understood as the node where both plurality and uniqueness emerge. The human condition of plurality is realized through action and, at the same time, it is through action that human beings express their distinctive uniqueness. Without action, then, we would not be human at all. For Arendt, being human and being a beginner who accomplishes their beginning through action in a public space are in fact one and the same. When the goals and purpose of action are already fixed and there is no room for change, detour, or further development, action loses its specific meaning, regardless of how lofty or noble those goals are and what authority dictates those goals. This is because the meaning of action also lies in addressing and bringing forth the new, and education reflects precisely such a broad and sustained commitment to the new. Through their capacity to act, human beings fulfill the implicit promise made at their birth: “The new beginning inherent in birth can make itself felt in the world only because the newcomer possesses the capacity of beginning something anew, that is, of acting” (ibid., p. 9).

Next, I wish to highlight that the “capacity of beginning something anew”, its “character of startling unexpectedness” (ibid., pp. 177–178) is not something added to human beings, or something we as humans may or may not accomplish. As humans, we come into the world as “*initium*, newcomers, and beginners” (ibid., p. 177). As Arendt states in *The Crisis in Education*, acting “for the sake of what is new and revolutionary” (Arendt 1977/1961, p. 189) for “the infinitely improbable which actually constitutes the very texture of everything we call real” (ibid., p. 170) is the first aim of education. Thus, the “character of startling unexpectedness” seems to me a wonderful image of the entanglement between living and education.

Such an understanding comes to reframe the interpretation and construction of one’s identity. Here, we should bear in mind that, according to Arendt, the public and political space precedes and grounds individuality: as stated above, for Arendt the subject reveals itself through its actions and speech, activities one may pursue only when one is connected to other human beings in the public arena. It is then not just that we cannot know how our actions will be taken and understood by others, but rather that—more radically—through action, one discloses oneself to oneself. One comes to know who one is *when* acting and speaking, not before. Human beings thus come to know who they are through their ongoing engagement with others—in endless and ever-changing processes—of which the structure and aims come to the fore in concrete situations of life.

To clarify, the central point of Arendt’s understanding is that human beings’ uniqueness emerges from them revealing to others who they are through their actions and speech, while at the very same time revealing to themselves who they are. Therefore, we cannot know who we are before such a revelation occurs. The dimension of the educational community is thus the root from which both one’s being and one’s becoming can grow, changing in unpredictable ways, and accomplishing the “miracle” of natality.

### Teachers’ Statements as Seen Through Arendt

At this point, we ask the question as to what kind of teaching and educational community emerges from Giovanna’s and Davide’s words. In their cases, too, the lockdown interrupted the space-time of normal schooling—the former *praesens*, if you wish—thus producing the conditions by which a different *praesens* was allowed to emerge. Such a different *praesens*—with everyone in their own room with their belongings—rather than creating a sense of distance and alienation, created a new intimacy and nearness, in which private and public were not just mixed: private and public were unrecognizable by their own features. Moreover, in a sense, their very division was useless for understanding the context, because a whole new environment emerged. As Davide effectively said, “everything was new.” In such a new environment, connections themselves were caught in a new web of meanings which transcended them, thus creating a new community.

This newness was also a defining feature of Giovanna’s teaching. Although Giovanna’s response to the embarrassment and discomfort experienced was quite different from Davide’s, the outcome was similar. The intentional neglect of students that she enacted resulted in a new, engaging manner of teaching rather than a kind of solipsistic teaching. Students were more involved than ever; from their exclusion from the community and even of communication came the discovery of a new behavior toward both the topics being explained and the educational community. Giovanna, in a sense, was teaching in a gap. We then ask: what kind of terrain may come out from a gap, and what meaning may be built or found, given that, when teaching, we are always already in the presence of some meaning? To propose a tentative answer, I wish to focus on the pronouns used by Giovanna in her report. At the beginning of the interview, she reported: “I began to concentrate… I began to delve.” It is as if they had to exclude students to teach them at all. At the end of their interview they stated, with a smile of surprise, “we discovered a new way to teach.” Students, unintentionally imitating her actions, built a new community while building new actions for themselves; through online teaching emerged an environment of intimacy, which deeply transformed students’ receptivity to both teacher’s words and the emerging community. Davide’s sense of joy and discovery while teaching shows how teaching is an ongoing indication to that region in which education and community joyfully emerge, while Giovanna’s experience speaks of a sense of collective, ongoing delving into the topics being discussed. Their words bear witness to an educational experience in its own right, one in which a realm of joy, freedom, fulfillment, and learning may emerge. “The fact of natality” (Arendt 1998/1958, 246) and the “startling unexpectedness” of living (Arendt 1977/1961, pp. 177–178) are displayed in both Davide’s and Giovanna’s accounts. In this sense, education illuminates the territory between what is known and the open space of radical possibilities before us. It is exactly such an unpredictable space of pure, radical possibility that is worth exploring educationally.

## Conclusion

In this paper, I have examined the lived experiences of teachers during COVID-19 using philosophical thought and scientific literature, thus combining educational philosophy with empirical research. I have analyzed the emotional impact that the abrupt shift to online teaching had on teachers’ work and lives during the various stages of the lockdown. Heidegger’s and Arendt’s thoughts, with their commitment to living, “being-with-others,” and being “for the sake of others” (Heidegger 1982/1927, p. 296), on the one hand, and narrative and the public dimension of identity (Arendt 1998/1958), on the other, are essential to this project.

Attempting to delve deeper into Heidegger’s and Arendt’s works, we may note that throughout his career, Heidegger explicitly developed an understanding of selfhood as an endless transformation through and for freedom and responsibility (d’Agnese 2017). For Heidegger, the self is fully committed to the openness that arises from its being-in-the-world (Thomson 2013). In other words, the ethical call that frames Heidegger’s thinking from the beginning (Standish 1992), his notion of selfhood as endless transformation, and the close connection between freedom and responsibility that emerges from his thinking (Thomson 2001) are crucial to grasping the sense of commitment and change to which the teachers’ lived experiences testify.

Arendt, on the other hand, offers an account of the human condition with profound pedagogical consequences. One of the recurring themes in Arendt’s work is her critique of any already established account of the human condition. For Arendt, the human condition is always already beyond the thinking that attempts to grasp it. This is because human beings become what they are in perpetual and ever-changing processes whose structure and goals are defined in concrete life situations. In this sense, as argued above, classrooms and educational spaces are conceived as spaces where human life takes shape in all its facets, and not just as places where skills are acquired to meet a preconceived notion of what is valuable. What is to be achieved and what students must strive for should be the subject of constant discussion and transformation. Otherwise, we run the risk of enhancing a reification of humanity, for “nothing entitles us to assume that man has a nature or essence in the same sense as other things. In other words, if we have a nature or essence, then surely only a god could know and define it, and the first prerequisite would be that he be able to speak about a ‘who’ as though it were a ‘what’” (Arendt 1998/1958, p. 10).

What is highly significant from an educational perspective is that, in Arendt’s view, this “meaningfulness” comes into play only in the dimension of “togetherness.” There is no room for something like “a private meaning.” According to Arendt, meaningfulness may rise only when human beings are considered “in the plural.” Only by talking, moving, and acting in a shared space may we experience meaningfulness.

This condition (dare I say the ‘essence’ of human beings in Arendt’s view?) makes teachers unprotected and vulnerable from the very beginning. This vulnerability does not stem primarily from the fact that we do not control the results of our actions and speech; rather, it stems from the uncertainty about how those actions and speech will be interpreted by others. I reveal myself to myself and come to know myself only when I reveal myself to others. “Although nobody knows whom he reveals when he discloses himself in deed or word, he must be willing to risk the disclosure” (Arendt 1998/1958, p. 180). Revelation does not take place only when the world or others reveal themselves to us. The original form of revelation is the revelation of the subject to herself, and it is highly significant that such revelation can only take place in our togetherness, without which human life is simply not possible: “To live an entirely private life means above all to be deprived of things essential to a truly human life… The privation of privacy lies in the absence of others; as far as they are concerned, private man does not appear, and therefore it is as though he did not exist” (Arendt 1998/1958, p. 58).

What Giovanna’s and Davide’s words bear witness to is exactly such a primacy of the public dimension, which comes to frame the private: by forcefully sharing their private space, they gained a deeper meaning of and commitment toward their profession. Conversely, Gennaro’s, Silvia’s and Luigi’s experiences of fear, distress, and anguish seem to derive from a lack of relatedness. Luigi “had to call his wife just to speak with her and break that sense of anguish,” while Gennaro was “paralyzed, in panic” and could not “think at all.” Also, Giovanna and Davide learned to change their teaching practices during the pandemic; initially they were neither calm nor happy with online teaching. However, during the pandemic, they managed to rethink their gestures, giving a new, unexpected meaning to their professional life, while building a new educational community with and for their students. Gennaro, Silvia and Luigi, on the other hand, remained trapped in the spiral of their fears.

Thus, the experiences of joy and fear seem to point in different but consistent ways to two essential educational dimensions: change and relationship. Without these dimensions, education, as human living, is simply impossible. The teachers’ experiences also reveal that they, to use Heidegger’s account, were simultaneously thrown into change and connectedness as they co-created with students the change and connectedness they experienced during teaching. Teaching as a physical, vulnerable, yet powerful experience is what is revealed by observing and learning from teachers’ words and gestures. Teachers changed themselves as they offered the opportunity for change to their students; this is the meaning of an ever-changing educational community.

As a concluding remark, I would like to return to the question of what the combination of philosophical thought and ethnographic research can yield. Such an intertwining can led us to think beyond and outside our thinking and engage with the messiness of experience, with what may generate perplexity. The etymology of the word “perplexity” indicates much more than just something to be resolved or put in order. Perplexity is from the Latin *per-plexus*: *per*, which means with, through, throughout, during, by means of, around, and *plexus*, which means woven, intricate, obscure, even. Thus, when teaching we may be perplexed, namely, we are going through and by means of what is woven, dwelling in and around what is intricate (in classroom’s relationships, for example), attending to such a meaningful messiness, even when such a task is risky, uncertain and frustrating. This seems to be the ethical core of inquiring through philosophy, and this is especially necessary, I would say, in times of fear, insecurity, and constant restriction of free educational spaces. Therefore, the attempt I present, is also a political *act.*

## Footnotes

1 Statement collected during the course’s conversations.

2 Statement collected through in-depth interview.

3 Statement collected during the course’s conversations.

4 Statement collected during course conversations and in-depth interview.

5 Statement collected through in-depth interview.
